# Using natural language processing to explore differences in healthcare professionals’ language on Functional Neurological Disorder: a comparative topic and sentiment analysis study

**DOI:** 10.3389/fdgth.2025.1691724

**Published:** 2026-01-16

**Authors:** Md Shadab Mashuk, Yang Lu, Lana Y. H. Lai, Matthew Shardlow, Shumit Saha, Ashley Williams, Anna Lee, Sarah Lloyd, Rajiv Mohanraj, Daniela Di Basilio

**Affiliations:** 1 School of Science, Engineering & Environment, University of Salford, Salford, United Kingdom; 2 Department of Computer Science, Loughborough University, London, United Kingdom; 3 Division of Informatics, Imaging & Data Sciences, University of Manchester, Manchester, United Kingdom; 4 Department of Computing and Mathematics, Manchester Metropolitan University, Manchester, United Kingdom; 5 Department of Biomedical Data Science, Meharry Medical College, Nashville, TN, United States; 6 Salford Royal NHS Foundation Trust, Northern Care Alliance, Salford, United Kingdom; 7 Division of Neuroscience, University of Manchester, Manchester, United Kingdom; 8 Faculty of Health and Medicine, Lancaster University, Lancaster, United Kingdom

**Keywords:** electronic health records (EHRs), Functional Neurological Disorder (FND), healthcare professionals, natural language processing (NLP), sentiment analysis, topic modelling, healthcare text analytics, machine learning

## Abstract

**Background:**

Effective communication is essential for delivering quality healthcare, particularly for individuals with Functional Neurological Disorders (FND), who are often subject to misdiagnosis and stigmatising language that implies symptom fabrication. Variability in communication styles among healthcare professionals may contribute to these challenges, affecting patient understanding and care outcomes.

**Methods:**

This study employed natural language processing (NLP) to analyse clinician-to-clinician and clinician-to-patient communication regarding FND. A total of 869 electronic health records (EHRs) were examined to assess differences in language use and emotional tone across various professionals—specifically, neurologists and psychologists—and different document types, such as discharge summaries and letters to general practitioners (GPs). Latent Dirichlet Allocation (LDA) topic modelling and two complementary sentiment models (VADER and Flair) were applied to the corpus. Sentiment analysis was also applied to evaluate the emotional tone of communications.

**Results:**

Findings revealed distinct communication patterns between neurologists and psychologists. Psychologists frequently used terms related to subjective experiences, such as “trauma” and “awareness,” aiming to help patients understand their diagnosis. In contrast, neurologists focused on medicalised narratives, emphasising symptoms like “seizures” and clinical interventions, including assessment (“telemetry”) and treatment (“medication”). Sentiment analysis indicated that psychologists tended to use more positive and proactive language, whereas neurologists generally adopted a neutral or cautious tone.

**Conclusions:**

These findings highlight differences in communication styles and emotional tones among professionals involved in FND care. The study underscores the importance of fostering integrated, multidisciplinary care pathways and developing standardised guidelines for clinical terminology in FND to improve communication and patient outcomes. Future research should explore how these communication patterns influence patient experiences and treatment adherence.

## 1 Introduction

### 1.1 Functional neurological disorder (FND) and the importance of clinician-patient communication

Functional Neurological Disorder (FND) is a neurological condition caused by a functional rather than a structural disorder, i.e., by changes in the brain network, rather than in the brain structure (1, 2). Symptoms of FND, which include tremor, paralysis, dystonia, sensory disturbances, speech difficulties, and dissociative seizures, are genuine and often likely to interfere with how a person functions and copes with daily life (1, 3). The burden of this disease is remarkable as FND is associated with high rates of distress and disability (2, 4–6) and a substantial reduction in quality of life, as well as impairments across multiple life domains, such as employment, socialisation, ability to be independent and experience of social stigma (7–9).

Furthermore, evidence indicates that FND is the second most common reason for neurological consultations after headaches (6, 10) and is linked to substantial healthcare costs for both individuals and services (11), with estimated costs exceeding US$1 billion/year in the United States (12) and £3 billion/year in England alone (13). Healthcare costs may be further enhanced by the frequent misdiagnosis of this disorder (14) and an average diagnostic delay of 7.2 years (15), which contributed to FND being labelled as medicine's “silent epidemic,” psychiatry's “blind spot” and a “demonised diagnosis” (16–19).

Fundamental to an individual's understanding and management of their FND diagnosis is the communication and therapeutic relationship with healthcare professionals (20, 21). Literature on FND highlighted that relationships with clinical professionals can, at times, entail negative interactions with FND patients (22–25). More specifically, studies on this topic highlighted that healthcare professionals in different roles, e.g., neurologists, general practitioners (GPs) and nurses, often express stigmatising views of FND patients as feigning their symptoms and pretending not to have control over them (26, 27). Additionally, they can perceive FND patients as “difficult” and frustrating, which in turn can lead patients to feel misunderstood, disbelieved, and rejected by their physicians (22).

Professionals' negative attitudes are often conveyed via the language they use in routine interactions with FND patients, and some studies shed light on professionals' use of stigmatising language concerning FND, including terms such as “fake” or “hysterical seizure” (27) and disparaging phrases such as “it's all in your head” (16). A recent review (28) reported how common ways used by healthcare professionals to describe individuals with FND included “attention-seeking”, “manipulative”, “annoying”, “impossible to help”, “troublesome”, “challenging”, and “frustrating” (13, 25, 26, 29–31). In contrast, FND patients reported being made to feel as if they were feigning or exaggerating their symptoms (32, 33). This highlights the need to develop a shared consensus on FND terminologies through standardising vocabulary across clinical professionals, in order to improve patient-clinician communication and reduce patient stigmatisation (34–36).

The necessity for shared vocabularies supporting multidisciplinary work in FND is also underscored by existing literature, which reveals both distinct and overlapping realms of expertise between the two types of professionals most frequently involved with the treatment of FND patients, i.e., neurologists and psychologists (37, 38). Neurologists' proficiency in diagnosing FND and identifying its symptoms through its distinguishing physiological features is well-documented (38, 39). Psychologists, conversely, are often described in FND literature as focusing on the more intrinsic “root factors”, behaviours, personality traits and psychosocial sequelae that are linked to FND (40). The need for complex, multidisciplinary approaches to treating FND has often been outlined as necessary (41, 42), although challenges persist in adopting clear, standardised guidelines to communicate with patients and in sharing information across services and healthcare professionals (43, 44).

One key factor in building collaborative, multidisciplinary approaches lies in the need to analyse the language used by healthcare professionals who support people with FND, to understand their vocabulary and the “emotional tone” underpinning their communications. This would allow for a better understanding of their core beliefs and conceptualisation of FND, and how these might influence both their relationships with patients and the overall quality of the support offered.

### 1.2 The application of artificial intelligence (AI) and natural language processing (NLP) to the analysis of clinician-patient interactions

Natural Language Processing (NLP) is a technique within the broader field of Artificial Intelligence (AI) that draws from linguistics and machine learning and aims at quantifying written language as vectors that can be statistically evaluated. It offers to broaden the reach of computational analysis to include human experience, emotion, and relationships (45, 46), and its application in the mental health domain has broadened exponentially in the past two decades (47–50). NLP is commonly applied to electronic health records (EHRs) to process large quantities of unstructured (human-authored) text, in order to return information about different text aspects, such as syntactic processing, semantic analysis (e.g., capturing meanings from single words or groups of words), and detecting relationships among terms and concepts (51–54).

Electronic health records are a rich source of data in the analysis and treatment of patients (55, 56), and NLP has proven to be effective in analysing and extracting information from clinical text data (57–59). NLP applied to EHRs might be particularly useful in exploring health professionals' vocabularies, as written text is likely to reflect the specific variations in their knowledge and expertise and their use of lay and professional vocabularies (58). NLP analysis on healthcare professionals' vocabularies has been applied in a variety of settings, for example, to explore and bridge the gap in vocabularies between healthcare professionals and their patients as well as people looking for health information online (60–63).

A specific NLP-related technique, known as “topic modelling”, has been increasingly used over the past few years to analyse textual data in EHRs to identify recurrent keywords and discussion themes (64) and identify people's perspectives on their mental health issues (65). Increasing interest in the healthcare domain has also been given to another NLP-derived technique, sentiment analysis, which has also been utilised to understand the general tone and emotion of clinical narratives (66). In other studies (67), EHRs such as hospital discharge notes were analysed to determine potential readmission and mortality risk from the “sentiment” of the notes, highlighting the multifaceted potential value of capturing “emotional tones” from EHRs to inform improvement in care pathways.

### 1.3 Identifying gaps and opportunities

While existing studies highlight the potential of NLP in healthcare research, its role in the FND domain has received little consideration. Moreover, the comparative analysis of professional discourse between neurologists and psychologists on FND through topic modelling and sentiment analysis remains unexplored. By applying these techniques to written documents produced by neurologists and psychologists supporting patients with FND, the present study sought to understand how different professionals communicate about and perceive FND. In turn, understanding clinical narratives, as well as their differences across care professionals, can inform training, clinical practice, and interdisciplinary collaboration, ultimately fostering a healthcare environment where the nuances of FND are more deeply understood and addressed with greater effectiveness. Prior NLP studies of EHRs have demonstrated how computational analyses of clinical narratives can expose patterns of documentation, framing, and implicit bias. For instance, Himmelstein, Bates, and Zhou (68) analysed 48,651 admission notes from a large US hospital system and identified that approximately 2.5% contained explicitly stigmatising terms such as “noncompliant,” “belligerent,” or “addict.” They further found that notes about non-Hispanic Black patients were significantly more likely to include such language compared with those about non-Hispanic White patients (adjusted probability difference = 0.67 percentage points), suggesting systematic linguistic disparities in clinician documentation. Complementary work by Weiner et al. (69) examined around 600,000 notes related to substance use disorder and found that stigmatising descriptors appeared in 16% of encounters, with disproportionate prevalence among minoritised groups and those receiving emergency care. A broader synthesis by Barcelona et al. (70) reviewed emerging computational approaches to detect bias and stigma in clinical notes, concluding that lexical cues and narrative framing in EHRs may perpetuate inequities through clinician perception and communication.

Beyond lexicon screens, topic modelling has been used to recover latent thematic structures within documentation. For instance, Sun et al. (71) applied topic modelling to ∼0.95 million clinical social-work notes, identifying 11 robust topics spanning social determinants of health (e.g., financial stress, abuse history, social support, end-of-life risks), thereby mapping how professional role and setting shape the themes clinicians document.

Sentiment-oriented analyses of EHR text have further shown that lexical tone can capture clinician attitudes or affective framing. A scoping review by Denecke and Reichenpfader (66) summarised more than 40 studies applying sentiment analysis to clinical narratives, highlighting that negative sentiment frequently correlates with deteriorating prognosis discussions, while positive tone aligns with counselling, discharge, or therapeutic engagement notes.

Together, these studies illustrate that (i) topic modelling can uncover implicit thematic emphases and conceptual framing within documentation, (ii) sentiment analysis can capture stylistic or attitudinal dimensions of clinician writing, and (iii) both approaches can reveal communication disparities associated with specialty, patient demographics, and clinical context.

Our study builds directly on this body of work by examining documentation related to FND—a condition largely absent from prior EHR/NLP investigations. We extend earlier approaches through a profession-comparative design, analysing correspondence authored by neurologists and psychologists to characterise differences in discourse content (via LDA topic modelling) and tone (via dual sentiment models: VADER and Flair). By jointly modelling thematic and affective dimensions, we aim to illuminate how disciplinary orientation shapes the way FND is linguistically represented within routine clinical documentation.

To the authors' knowledge, this is the first study to employ well-known NLP techniques for a comparative analysis of the narratives and underlying emotional tones of different healthcare professionals working in FND care.

## 2 Methodology

The current study aimed to leverage the potential of NLP to conduct an in-depth analysis of the vocabularies used and emotional tones expressed by different groups of healthcare professionals supporting individuals with FND. Two NLP techniques, topic modelling and sentiment analysis, were used to extract and classify electronic clinical documents from the outpatient services of the Salford Royal Hospital (Northern Care Alliance NHS Group Trust), a large hospital located in the Northwest of the UK. Ethical approval to extract clinical documents from hospital records was obtained from the R&I department of the Northern Care Alliance NHS Group Trust (approval number 22HIP47).

### 2.1 Data source

Clinical correspondence authored between 2011 and 2022 was retrieved from the Salford Royal Hospital EHR system. The search targeted outpatient letters and reports associated with confirmed or suspected Functional Neurological Disorder (FND) using a combined query of diagnostic codes (ICD-10: F44.x, R56.8, with G40.x exclusions) and free-text filters for relevant keywords such as “*functional neurological”*, “*non-epileptic attack”*, “*psychogenic seizure”*, and “*PNES”*. This broad strategy was designed to capture all documents that could reasonably pertain to FND across clinical services and yielded 1,027 candidate records. Each document was then manually screened by four members of the research team [two research assistants [SL, AL], a clinical psychologist [DDB], and a neurologist [RM]] according to pre-specified inclusion and exclusion criteria. To ensure consistency, reviewers applied a structured checklist that operationalised “completeness” and “quality” for the purposes of NLP preprocessing. Documents were retained if they explicitly discussed or diagnosed FND or functional symptoms, contained at least 200 words of narrative text, included an intact and legible body of text without scanning artefacts, truncation, or corrupted formatting, and passed anonymisation checks. Records were excluded if they contained substantial redaction errors, illegible sections, missing pages, or insufficient clinical content to support meaningful computational analysis. Duplicate or near-duplicate records—identified through matching dates, authors, or recipients—were removed. All documents were independently reviewed by two screeners, and any uncertainties or disagreements were resolved through discussion with the wider clinical team to reach consensus. Because decisions were based on explicit rule-based criteria rather than subjective rating scales, formal inter-rater reliability statistics were not calculated.

Application of these criteria resulted in a final corpus of 978 eligible documents authored by neurologists (*n* = 550), psychologists (*n* = 319), and other professionals (109) such as general practitioners, physiotherapists, and pain specialists.

All documents were manually anonymised by research assistants [SL and AL] employed at the Salford Royal Hospital, by removing any identifiable information before commencement of study. The date, type of document (i.e., letter type), professional group category (i.e., neurologists, psychologists or others) and raw unstructured text were preserved and provided as a Word file to researchers. Assignment of documents to provider specialty groups was based primarily on the author's information contained in the metadata and signature block of each clinical correspondence. Each document in the EHR system specifies the clinician who authored and signed the note, along with their professional designation (e.g., Consultant Neurologist, Clinical Psychologist) and associated outpatient service. When the signature block alone was insufficient—for example, where only initials or generic titles such as “Consultant” were shown—departmental metadata (clinic location, service line, and appointment type) were used to confirm the author's specialty. Documents were attributed to a single specialty based on the author of record; the presence of multiple professionals mentioned in the text (e.g., copied recipients) did not influence specialty classification. On rare occasions where two specialties were jointly involved in a visit, the author listed as the primary signatory was used as the determinant. Specialty assignment was not provider-static but document-specific: clinicians were classified according to the role associated with each individual letter rather than globally across all records. This ensured that specialty attribution accurately reflected the clinical context and authorship of each document.

A Python script was written to parse the document, retrieve, and store the relevant information of each letter into a CSV file, and create a structured corpus containing the “document type”, “professional group”, “date” and “text”. These attributes were obtained directly from the structured metadata provided in the EHR export (e.g., author designation, department, appointment type, and document category), rather than inferred from text. The parsing script was therefore responsible only for extracting the narrative body of each document. Because outpatient letters at this Trust follow a semi-standardised format (with consistent signature blocks and header fields), rule-based pattern matching (e.g., detecting section delimiters such as “Dear…”, “Yours sincerely,” or department banners) was used to isolate the main free-text narrative. Standard headers, templates, and administrative boilerplate were removed so that only clinician-authored prose was retained for analysis. To assess accuracy, a random 10% subsample of documents was manually compared against the raw files, confirming >95% agreement between the parsed output and the original text.

The corpus was further cleaned for missing values, replacements, and misspelt medical terminologies, which were identified through manual observation. Documents were included if (i) they explicitly referred to FND, (ii) contained ≥200 words, (iii) were complete and legible, and (iv) passed anonymisation. Two reviewers [AL and SL] screened independently; discrepancies were resolved by a third reviewer [DDB].

Of the 978 documents identified for analysis, 109 labelled as “Other”, were excluded from the analysis. The total word count per document ranged between 200 and 1,200 words. Focus was given to the labelled professional groups (i.e., neurologists and psychologists), giving a total sample size of 869 documents. The documents were also categorised by type, i.e., referrals, clinic letters, GP letters, assessments, and discharge letters. Details of the different letter types and contents are provided in Supplementary Appendix 1.

### 2.2 Framework of exploratory analysis

**Figure 1:**
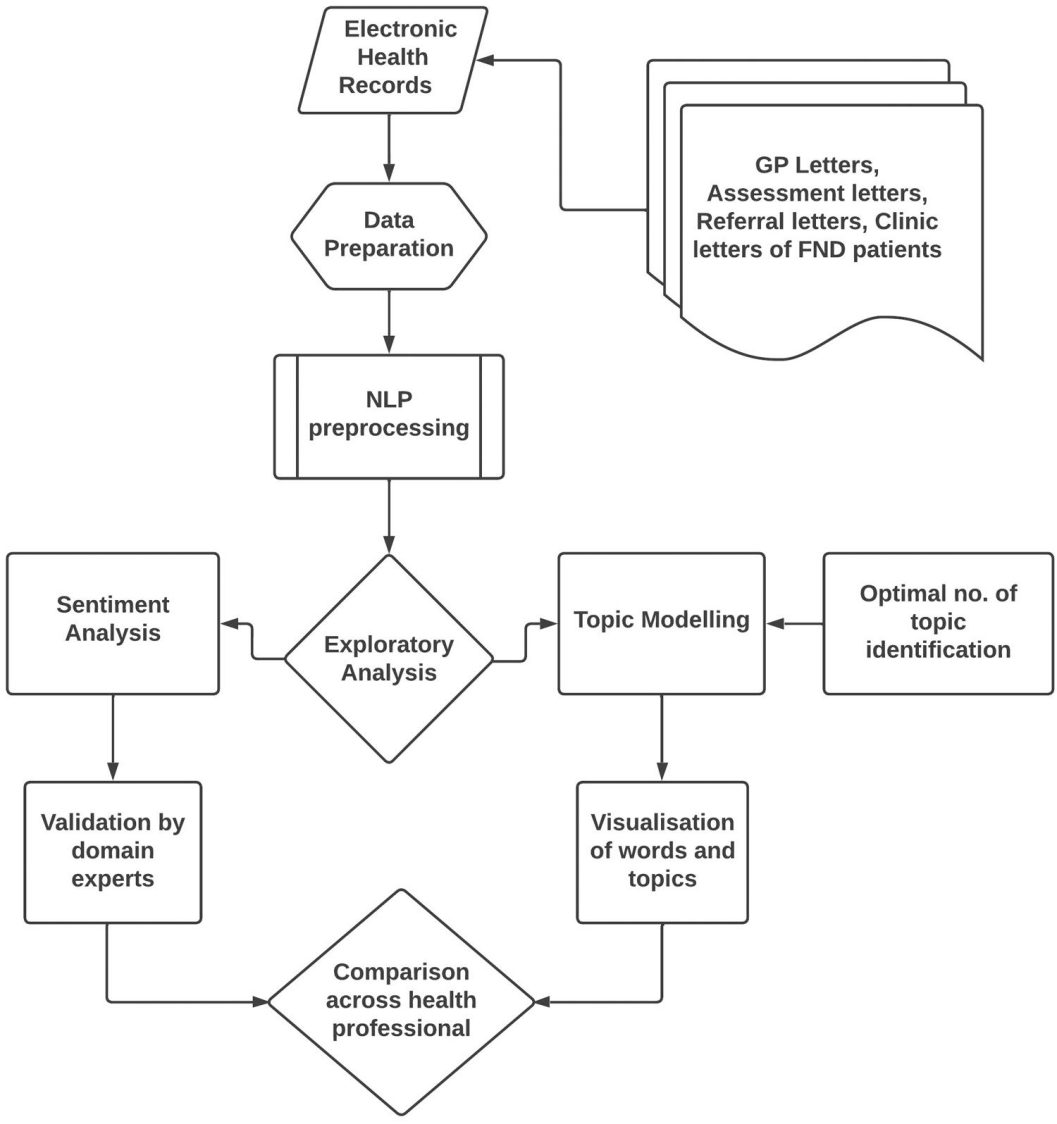
A process flow diagram showing the pipeline of the exploratory analysis of the medical records.

In this section, the general framework of the exploratory analysis is presented in Figure 1. In addition to the steps discussed above, NLP preprocessing steps such as tokenisation, bigrams, trigrams, lemmatisation and removal of stop words were also applied. Then, the established topic modelling and sentiment analysis techniques were used to identify differences in the usage of words, topics of discussion, and tone/sentiment across healthcare professionals (i.e., psychologists and neurologists), as well as by document types.

#### 2.2.1 Preprocessing

To identify the topic of discussion, preprocessing of the data was carried out (Figure 1). The stages of data processing included the following:

1. Tokenisation; Texts are lower cased and NLTK's (72) word tokenise library was used to tokenise sentences into tokens of words. Custom tags such as titles (ms, mrs, mr, miss), date and time, special characters and a minimum word length (4) were also applied to ensure that only the most relevant tokens were left. Parts of speech (POS) tags (NOUN) were also applied. Noun-only POS tags were used as we were only interested in identifying important keywords.
2. Ngrams; Bigram and Trigram models were also created and applied using Gensim's (73) Phrases library for any tokens that can be combined as bigram or trigram.
3. Lemmatisation; Lemmatisation was applied using NLTK's wordnet/morphy library as appropriate.
4. Stop words; Stop words were removed using Gensim's simple preprocess library.
5. To further optimise the quality of topic keywords, some high-frequency common terms widely used by both professional groups, such as “diagnosis”, “session”, “therapy”, “service”, “attack”, and “episode” found across both psychologists’ and neurologists’ records were removed during tokenisation. This was done based on empirical observation by running the topic model several times and identifying keywords that commonly occurred within the topics. The clean data was then passed on to the topic modelling algorithm.

During preprocessing topic modelling, we retained only nouns and noun-phrases to focus on stable semantic entities (e.g., *symptom, diagnosis, treatment, patient*) that define the conceptual structure of the documents. This approach reduces lexical noise and improves the interpretability of topics by emphasising recurring clinical concepts rather than variable grammatical or stylistic features. However, we acknowledge that adjectives and adverbs can carry important evaluative or affective information (e.g., “*severe seizure,” “gradual improvement”*). To preserve this nuance, the complete lexical set—including adjectives, adverbs, and verbs—was retained for the sentiment analysis.

All sentiment and topic modelling analyses were performed on the full narrative body of the letter, excluding only fixed headers, signatures, and administrative fields. No restriction was applied to specific clinical sections (e.g., “Assessment” or “Plan”); instead, all clinician-generated free text was analysed to capture the full communicative tone and thematic content. Common template headings (e.g., “Assessment and Plan”, clinic labels, service identifiers) were automatically removed to avoid inflating word frequencies from non-semantic boilerplate. Terms such as “assessment”, “neuropsychology”, or “clinic” that survived this filtering reflect their use in the narrative body of the document rather than their appearance in standardised header fields.

#### 2.2.2 Topic modeling

To understand the differences in how psychologists and neurologists approach FND patients, it is important to identify common discussion topics and the words associated with each topic. To do so, topic modelling, a form of computational text mining based on word co-occurrence within a corpus, was used. Topic modelling algorithms take a collection of documents as input, discover recurring “themes” discussed within the collection (topics), and then determine the degree to which each document presents each of the topics identified (74). One of the most widely used unsupervised document modelling techniques, Latent Dirichlet Allocation (LDA), was applied. LDA is a generative probabilistic Bayesian model providing a representation of a document in the form of topics and topic probabilities, and it is claimed to be the simplest and most widely used topic modeling technique in fields outside of computer science (74, 75). Further details related to the underlying mathematical principles can be found in the original paper (76).

**Figure 2:**
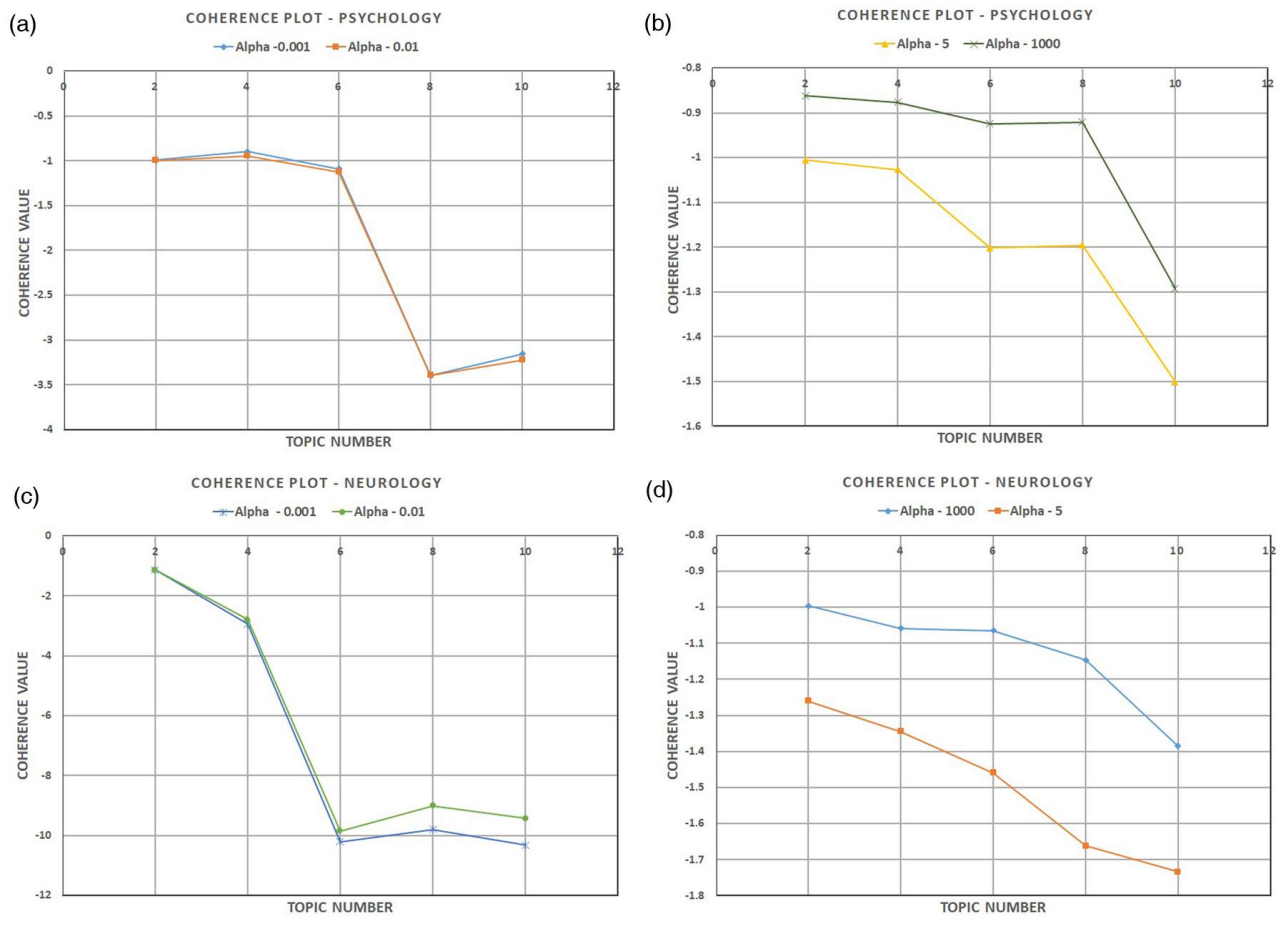
Coherence plots for different values of alpha for psychology and neurology data.

In our study, Gensim's LDA model for Python was used to generate the topics of discussion. Before generating the topics, the optimal topic number was estimated by tuning the hyperparameter alpha (*α*), which helps to determine how the topics will be distributed across the document corpus. A higher *α* meant a high number of topics generated but sparsely distributed across the documents, whereas a lower alpha meant a smaller number of topics but more focused and specialised. To determine the optimal topic number, α was varied for a limited range of values to determine how the topic coherence value (CV) (77), which determines the quality of topics generated, changed across the psychologists' and neurologists' datasets. The topic number was varied between 10 and 2, and the following alpha values were experimented with; (0.001, 0.01, 5, 1,000) to analyse the CV score for each topic number as seen in Figure 2.

Figure 2 shows that the CV score started with a value closer to zero before falling sharply approximately between topics 6 and 4 and then flattening out for *α* = 0.001 and 0.01 for psychology and neurology records, respectively. The higher coherence value signifies dominance and importance of topic within the analysed data set. When *α* = 5, the coherence plot does not show a clear distinction between dominant topics and shows a gradual fall; similarly, when *α* = 1,000, it again shows a reasonably gradual fall from topic 8 onward. In addition, the coherence value is close to zero for the neurology and psychology data when *α* = 1,000, but they do not seem to be converging quickly enough, as is the case with *α* = 5 values. As such, there is a need to achieve a right balance between the topic distribution not being too widely spread across the document, and a topic number which has a coherence value reasonably closer to zero. Hence, it can be concluded that it is reasonable and safe to decide that the optimal topic number for this study should be five, as it is more dominant and coherent in both the neurology and psychology datasets and also choose the value *α* = 0.01 as the optimal value for creating the final model. Final modelling parameter value details can be found in Supplementary Appendix 7.

As last step, the interpretation and labelling of topics generated by the LDA models were carried out by two members of the research team: DDB (clinical psychologist) and a scientist (MSM). Each reviewer independently examined the top-ranked terms and representative documents associated with each topic and proposed descriptive labels based on semantic coherence and contextual meaning. The review focused on assessing whether the high-probability words within each topic formed an interpretable and clinically meaningful theme, taking into account the professional context of the documents. To preserve the quality of the topic distribution, term removal was guided by explicit rules rather than discretionary judgement. “Quality” in this context referred to the semantic coherence, interpretability, and distinctiveness of topic clusters produced by LDA. Prior to final modelling, several pilot LDA runs were conducted to identify extremely high-frequency terms that dominated topics but contributed minimal semantic information (e.g., generic administrative words such as “session”, “service”, “letter”, or ubiquitous clinical markers such as “diagnosis” or “attack”). These terms were removed only when they (i) appeared across both professional corpora at similarly high frequencies, (ii) carried no discriminative value for understanding clinical discourse, and (iii) reduced topic coherence by inflating generic clusters. No profession-specific or content-bearing clinical terms were removed. The same removal rules and list of terms were applied symmetrically to the neurology and psychology corpora to avoid introducing group-specific artefacts. Topic coherence metrics and keyword distributions were re-evaluated after each removal step to ensure that selective term removal did not artificially accentuate differences between groups.

This process was semantic and interpretive in nature, reflecting the exploratory aims of the study rather than a hypothesis-driven coding framework. For this reason, no formal inter-rater reliability coefficient (e.g., Cohen's *κ*) was calculated. Instead, differences in interpretation were discussed collaboratively until a shared understanding of the topic structure was reached. Topic evaluation followed an interpretive review process in which three team members independently assessed the semantic coherence and clinical relevance of each topic; only topics judged to represent meaningful and interpretable themes were highlighted in the results tables. This approach ensured that both computational and clinical perspectives informed the final topic labelling, enhancing conceptual validity and interpretability while maintaining methodological transparency. After model training, a structured interpretive review was conducted to evaluate and label the topics generated by LDA. This process was carried out by three members of the research team: a clinical psychologist (DDB), a neurologist (RM), and a data scientist (MSM), each bringing complementary domain knowledge relevant to FND and computational text analysis. Reviewers independently examined (i) the top-ranked keywords defining each topic and (ii) representative documents and text excerpts most strongly associated with each topic (based on per-document topic probabilities). All texts were reviewed in the same pre-processed form that entered the LDA model to ensure alignment between model outputs and human interpretation. Following an independent review, the team met to compare interpretations, document points of convergence or disagreement, and agreed on descriptive labels for each topic. This procedure ensured that topic naming and relevance assessment were transparent, systematic, and grounded in both computational evidence and clinical-contextual understanding, and it aligns with mixed-methods topic-modelling practices increasingly recommended in psychology and health informatics, where model-derived topics are routinely subjected to structured human evaluation of coherence and meaning (78). Similar combinations of automated topic discovery and expert review have been used to assess the interpretability of topics learned from primary-care clinical reports, clinical social-work and oncology notes, and mental-health or therapy-related texts, where clinicians or trained researchers examine keyword lists and representative documents to label topics and discard non-informative or purely administrative themes (71, 79–81).

#### 2.2.3 Sentiment analysis

Sentiment analysis is a computational method for determining the emotional tone behind words. It is essential for understanding the attitudes, opinions, and emotions expressed in textual data, and has been increasingly applied in clinical informatics to characterise tone, evaluative language, and communicative stance within medical narratives, including referral letters, progress notes, and discharge summaries (82). Prior work shows that affective and evaluative language can shape how clinicians frame symptoms, signal diagnostic certainty, and communicate levels of concern or reassurance. On this basis, sentiment analysis provides a theoretically and empirically motivated tool for examining narrative content in FND-related correspondence.

In this study, sentiment analysis was conducted to explore the sentiment levels of neurologists and psychologists as reflected in various types of clinical documentation and to verify the topics and themes of discussion observed from the topic modelling analysis. Two pre-trained sentiment models, VADER (83) and Flair (84), were implemented. VADER is a lexicon and rule-based sentiment analysis tool (83) deployed to assess sentiment levels across different file types within the clinical documentation of each professional group. Its efficiency and sensitivity to subtle linguistic cues make it particularly suitable for analysing the structured format and concise language often found in such documents. However, it does not examine the context-dependent nuances of the sentences. Therefore, while the VADER's lexicon-based tool may be fast and effective for processing large volumes of documents, it may not fully capture the context-dependent nuances of sentiment. Flair's deep learning approach fills this gap by analysing sentences in their entirety, considering the context to provide a nuanced understanding of sentiment. This is crucial for understanding complex narratives found in referral letters or discharge summaries, where the sentiment may be influenced by medical conditions, treatment outcomes, or patient experiences. Previous work in clinical informatics (66, 70, 85) has shown that sentiment and evaluative language in clinical narratives—including progress notes, discharge summaries, and other EHR text—can capture aspects of clinician attitude, framing, or stigma and can be associated with care processes or outcomes. On this basis, we treat sentiment analysis as an exploratory tool to characterise the affective tone of FND-related correspondence (e.g., referral letters and discharge summaries), with implications further developed in the Discussion, rather than being assumed *a priori*.

Additionally, clinical documents pose a unique challenge due to their specialised medical terminology. Together, these models offer a comprehensive sentiment analysis that can be used to cross-validate each other's outputs, since they are not specifically trained on medical terminology. This tailored application can enhance the reliability of sentiment analysis and contribute valuable perspectives on the emotional and professional dynamics within clinical settings.

#### 2.2.4 Sentiment contributors and phrase-level interpretation

As automated sentiment models can sometimes misread the tone of clinical writing, an expert review was conducted to evaluate how accurately the AI had captured the emotional and relational nuances of clinician–patient language. The two sentiment models (VADER and Flair) assigned each document a polarity score based on the words and expressions it contained. To understand what drove these scores—and to judge the reliability of the AI interpretations—we identified the top-ranked positive and negative contributors in each professional group and reviewed them manually.

This review was carried out by a research team member (DDB) with clinical expertise but not directly involved in the care of FND patients. This composition allowed us to balance clinical insight with objectivity: DDB's familiarity with healthcare documentation ensured accurate interpretation of clinical terminology, while her lack of direct involvement with FND cases reduced the likelihood of interpretive bias arising from personal clinical experience in this specific domain.

The main task was to evaluate whether the AI's sentiment assessment was consistent with the intended clinical meaning of the text. This clinician-driven interpretation was essential for determining not only how reliable and accurate the AI-generated sentiment scores were but also why certain language appeared more positive or negative. Many of the words that increased negative sentiment were, in fact, clinically descriptive rather than judgmental, for example, phrases such as “frequent attacks,” “poor sleep,” “limited progress,” or “increasing seizure frequency.” These reflect the patient's clinical status rather than negative clinician attitudes. In contrast, a smaller number of expressions carried evaluative or interpersonal overtones, such as “noncompliant,” “attention seeking,” or “difficult patient,” which can suggest implicit stigma or frustration. Positive sentiment, by contrast, was often linked to collaborative or improvement-oriented language, such as “engaged in therapy,” “coping better,” and “showing progress.”

This clinician-guided review confirmed that most of the sentiment variation across professional groups was grounded in clinical focus rather than bias, while also highlighting how occasional evaluative language may influence tone. It provided a critical check on the accuracy of AI-driven sentiment analysis and ensured that interpretations remained sensitive to the realities of clinical communication.

To support interpretation of sentiment outputs, we conducted a structured contextual review of exemplar sentences and local text segments associated with high-impact sentiment values. For salient terms (e.g., “friend”), reviewers examined the immediate sentence and its surrounding context within the pre-processed text passed to VADER and Flair, in order to determine whether usage reflected interpersonal tone, social history, third-party reporting, or routine clinical documentation. This procedure was not designed to infer stylistic categories but to ensure that sentiment scores aligned with the lexical meaning of terms in context. Such contextual inspection is consistent with best practice in clinical NLP, where sentiment tools identify valence rather than interpersonal tone and where negative polarity often reflects symptom negation, risk communication, or acute-event documentation rather than affective stance (82, 86).

## 3 Results

### 3.1 Topic modeling

The topic analysis was divided into two stages. In the first stage, words and themes of topic discussions between psychologists and neurologists were compared. In the second stage, a detailed analysis was conducted on psychology and neurology records according to letter types. Since LDA is stochastic, rather than training only one topic model on the entire corpus, separate topic models were trained for each psychology and neurology document set separately to ensure only topics from the relevant documents were generated, with no overlap across the two professional groups. This ensures that any differences observed in the generated topic will be based only on the type of notes analysed. The same approach is applied when analysing topics across document types (i.e., referrals, clinic letters, GP letters, assessments and discharge letters) for both the professional groups considered (psychologists and neurologists).

#### 3.1.1 Professional group comparison: identifying main differences in communication between neurologists and psychologists

**Figure 3:**
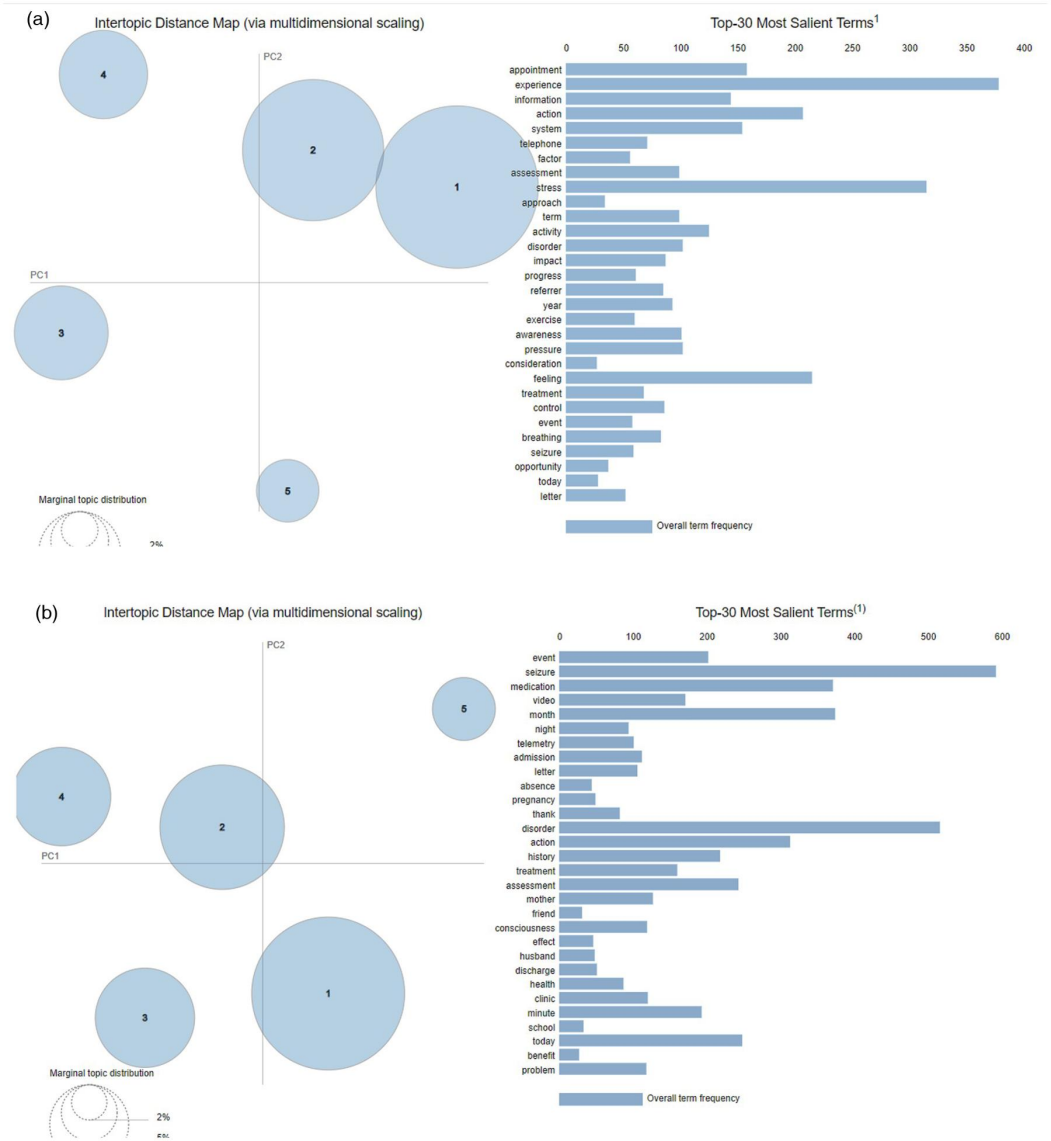
**(a)** Topic distribution plot for all psychology data. **(b)** Topic distribution plot for all neurology data.

Firstly, the main differences between the two professional groups were considered across psychology and neurology documents (regardless of the document letter type—e.g., discharge letter). Figures 3a,b below show the most salient terms used by neurology and psychology professionals in their respective document sets. Additionally, they also show inter-topic distribution plots, which demonstrate the “importance” of each topic (i.e., the frequency with which that topic was addressed in clinical documents). Both psychological and neurological medical records had evenly distributed and similar-sized topics of discussion, with some contributing less to the discussion. Most of the topics were distinct from one another, although there were some overlaps between topics 1 and 2 in psychology records.

The top thirty salient terms across all the psychology documents—as seen in the inter-topic distribution plots (Figure 3a above)—highlighted the frequent use of terms related to inner/subjective experiences of people with FND (e.g., “experience”, “stress” and “awareness”) and the emotional and psychological correlates of living with their symptoms [e.g., “feeling”, “impact”, “control” (or lack of thereof) and “pressure”]. It is also worth noticing that the most commonly used terms include positive and proactive language, such as “action”, “progress” and “opportunity”.

Overall, in psychology professionals' language, there seems to be a well-balanced importance given to patients' treatment (including aspects such as medication, care approaches and intervention planning) as well as more personal dimensions, such as the psychological sequelae of living with FND.

Table 1 below shows the important keywords for psychology documents based on topics of discussion. Although the topic modelling algorithm produced five topics, Table 1 only shows the four most relevant topics of discussion, as these were deemed to be the most representative of relevant aspects in clinicians' communication with and about patients. For each topic, some of the most relevant keywords are reported, together with a topic name based on the semantic commonalities among topic keywords. Topic relevance was determined using standard LDA interpretability criteria, including semantic coherence, clinical meaningfulness of the top-ranked keywords, and agreement among reviewers regarding whether a topic reflected a recognisable theme in the clinical discourse. All five topics generated by the model were examined; however, one topic was excluded from the summary tables because its highest-probability terms represented generic administrative or structural vocabulary without a coherent thematic focus, and therefore offered no clinically interpretable content. Importantly, this topic was not removed from the modelling process itself—only from presentation—ensuring that the comparative analysis across professional groups remained methodologically intact and free of selective reporting. Following the identification of topics through topic modelling, a human-led process of interpretation was applied to the topics retrieved as described in Section 2.2.2 above. As several authors pointed out, the topics retrieved by topic modelling techniques need to undergo a process of sense-making based on the researcher's understanding of words reported as strongly associated with each topic. To do this, an interpretation process was performed independently by the study principal investigator (MSM) and a researcher team member with clinical expertise (DDB). Interpretations and themes were compared, and discrepancies were resolved by consensus.

- Topic 1 (Psychoeducation about FND): The dominant terms revolved around discussions of factors and experiences related to seizures, access to information, and approaches to managing FND (e.g., adopting self-compassion, and taking care of one's mental and physical health).
- Topic 2 (Diagnosis and prognosis): This theme focused on the diagnostic process both from a medical perspective (with related keywords such as “assessment” and “disorder”) and from a more subjective one, with terms such as “stress” that suggest a consideration of how the diagnosis of FND can impact on people's wellbeing and personal experiences in different areas of their lives.
- Topic 3 (Pragmatic support): The dominant terms referred to objective ways of obtaining support for FND symptoms, including appointments, receiving clinical letters, adopting different strategies for treatment and using medication.
- Topic 4 (Emotional/personal support): Although similar to the previous topic in its focus on long-term management strategies (e.g., “management and ‘review’), this topic also seemed to stress a social and emotional component that may play a key role in coping with FND in the long term. Indeed, there were terms such as “family”, “emotion”, “sensation” and “feeling'” that seemed to refer to the importance of emphasising relational and emotional components of symptomatic management in FND.

**Table 1:** Keywords and topics: psychology documents.

Topic no.	Topic name	Selected keywords
1	Psychoeducation about FND	Approach, information, change, exercise, compassion, respect, health, body, trauma
2	Diagnosis and prognosis	Assessment, disorder, symptom, progress, stress, year, appointment, history, treatment
3	Pragmatic support for symptom management	Appointment, telephone, letter, strategy, treatment, support, health, medication, breathing
4	Emotional and social support for symptom management	Feeling, pressure, anxiety, management, family, emotion, sensation, awareness, communication

Neurologists' conversations, on the other hand, seemed to show different patterns in their communications with and about clients (Figure 3b). More specifically, the 30 most salient terms across all neurology documents showed that their discussion mostly revolved around seizure attacks, admission and potential actions taken during those episodes. The focus was also on patients' health and their treatment, history and medication, as well as the duration of attacks, due to using terms such as “month”, “night”, “minute”, and “today”. A closer look at the topic distribution in Table 2 showed topic-wise discussion themes mentioned by neurologists.

- Topic 1 (Assessment of FND and contributing/maintaining factors): The dominant theme revolved around discussions about the assessment of FND symptoms (“seizure” and “assessment”), medication, and characteristics of service provision (“e.g., admission” and “neuropsychology”).
- Topic 2 (Treatment planning)—The second theme placed an emphasis on strategies for ongoing monitoring aimed at establishing best treatment options (e.g., “telemetry”, “video”, “treatment” and “medication”) according to both patient symptoms (“seizure”) and other conditions (“pregnancy”). It also contained a reference to different stages of the patient journey, such as “admission” and “discharge”.
- Topic 3 (Management of FND symptoms)—Quite similar to the previous theme, this third theme focused on different aspects of managing FND, including taking “medication”, attending “appointments” and adopting strategies for ongoing “management” of this condition.
- Topic 4 (Recommendations for support and long-term management)—this theme included terms related to modifications in “behaviour” and “health” attitudes to manage FND symptoms in the long term. Interestingly, though, there were some terms related to key aspects that neurologists appear to consider as crucial in FND management: the involvement of patients’ family and close network (“father”, “husband” and “friend”), attention to the person overall mental health (“citalopram”) and psychological wellbeing (“awareness” and “disturbance”).

**Table 2:** Keywords and topics—neurology documents.

Neurology documents		
Topic no.	Topic name	Selected keywords
1	Assessment of FND and contributing/maintaining factors	Medication, seizure, assessment, patient, admission, management, clinic, stress, sleep, neuropsychology
2	Treatment planning	Seizure, telemetry, treatment, medication, admission, discharge
3	Management of FND symptoms	Medication, today, year, appointment, week, attendance, management
4	Recommendations for support and long-term management	Citalopram, behaviour, health, husband, disturbance, awareness, management

Following these analyses, a further set was performed to gain a more granular understanding of communication differences between the two professional groups (psychologists and neurologists) across different document types (referral letters, clinic letters, GP letters, assessment and discharge letters). The topics retrieved and the related most relevant keywords topic-wise are illustrated in Supplementary Appendices 2–6, and the main differences that emerged are presented descriptively below.

#### 3.1.2 Group comparisons across document “types”: exploring document types separately better to understand differences in communications between neurologists and psychologists

Once the main language differences between psychologists and neurologists had been outlined, our analyses progressed toward further investigating the granularity of these differences by analysing their communication patterns and topics, focusing on each document type (referral letters, clinic letters, GP letters, assessment letters, and discharge letters) separately.

##### 3.1.2.1 Referral letters

Both referral letters written by neurologists and by psychologists showed some commonly recurring topics, such as the reference to FND symptoms [“seizure”, (alteration in) “consciousness”] and mention of assessment, intervention planning and management of FND (“assessment”, “intervention”, “hospital”, “outpatient” and “clinic”). Another important commonality between the two groups is the reference to aetiological and maintaining factors of FND, such as “trauma” and “stress”).

The main differences between the two professional categories were reflected in the use of medical terminology (e.g., “disorder”), which was more frequent in neurologists' referral letters as compared to psychologists'. On this note, neurologists also seemed to refer more often to other medical conditions (e.g., “epilepsy” and “autism”, “drug” [abuse] comorbid to FND, that they may have considered and/or assessed for during patient visits. They also mentioned a broader list of FND-related medical terms, such as “convulsions”, [lack/loss of] “consciousness” and “myoclonus”.

Interestingly, psychologists, but not neurologists, mentioned that patients were usually women, therefore giving relevance to personal characteristics such as their gender. They also placed great importance on the overall quality of patients' lives by frequently paying attention to pain (“painkillers”, “headaches”) and quality of “sleep”. They also mentioned the term “goal”, which in their referral letters was often used to indicate the goals agreed upon with the patient and to achieve which a referral to another service might have been needed (e.g., psychological services offering trauma-focused therapy). Neurologists showed appreciation of the “burden” that FND can represent patients' lives and mentioned this term in a few of their referral letters, mostly to indicate the need and/or urgency for a patient to receive support.

##### 3.1.2.2 Clinic letters

The split between a “medical focus” vs. a more comprehensive attention to a range of factors influencing FND in the two professional groups is perhaps even more evident in the topics retrieved in the clinic letters. Whilst neurologists' discussions revolved around the “brain” and physiological manifestations of FND such as “myokymia”, “seizure” and “memory”, psychologists' vocabulary encompassed a range of terms that showcased their appreciation for different FND-related and personal aspects of people's lives. Among these, there were “childhood” experiences, “feeling[s] and emotions[s]” reported in clinical appointments, and an appreciation for the subjective experience of FND-related “stress”, “overwhelm” and “pressure” experienced by patients. There was also a reference to psychological correlates of FND, such as “dissociation”, and a mention of cognitive, emotional and behavioural coping strategies (e.g., “belief”, “habit”, “commitment”), representing a unique feature in psychologists' communication. Although terms such as “partners” and “family”—referring to personal dimensions of people's lives- were also present in neurologists' communication, these terms were used in a more factual/descriptive way, e.g., to describe the caregiver(s) accompanying patients to neurology appointments, rather than to convey an appreciation of patients' social/family network and the role they might play in FND management.

##### 3.1.2.3 GP letters

The trend described above (using a medical vocabulary vs. a more holistic one) was once again evident in the topics retrieved from letters sent to GPs. All the five topics that were more relevant in neurologists' communications with other medical professionals (GPs) contained a reference to symptoms, diagnostic and prognostic processes (“NEAD”, “recovery”) and types of support and services (e.g., “neurology” and “community” services, “letter” and “telephone”) that may be part of the patient journey. Words such as “friend” were present, but as above, they were mostly used to provide contextual information, as exemplified by the following excerpt from a neurologist's letter to a GP: “*Her friend (…) says that (…) was at her home and was about to sleep on her couch when she started staring into space*”.

##### 3.1.2.4 Assessment letters

The topics retrieved in assessment letters showed the different conceptualisations of the assessment processes in the two professional groups considered, with once again a medical and a biopsychosocial model emerging from clinicians' written communications. The primary dimensions considered in neurologists' assessments are physiological “signs” such as “tremor”, service engagement (“treatment”, “attendance”, “history”, “month”), medication (“lamotrigine”, “risperidone”) and comorbidities that may be present or warrant further exploration (“anxiety” and “depression”). The term “childhood”, indicating a consideration for early traumatic experiences influencing FND, was present but was retrieved as part of topic five, meaning its use was not so frequent. Conversely, psychologists' assessment letters offered proof of the importance of many factors when considering the best care options for patients. More specifically, a set of medical/physiological aspects was considered ('stress”, “eizure”, “medication”), but this was in conjunction with psychosocial aspects (“exercise”, “emotion”, “relaxation”, “awareness”) that showed how psychologists think of FND patients from a perspective of complexity, encompassing disorder-related dimensions but also opportunities for symptomatic management through engagement in activities that can improve psychosomatic wellbeing. In this regard, it was worth noticing the presence of positive and encouraging terms in their vocabulary, such as “opportunity”, “strategy” and “development”.

##### 3.1.2.5 Discharge letters

In line with previous considerations, the analysis of discharge letters evidenced the presence of a communicative approach in neurologists' written text that favoured objective/factual information to motivate patient discharge, as evidenced, for example, by salient terms composing topic one (e.g., “discharge”, “admission”, “neurology”, “assessment”, “gynaecology”, “medication”, “history”, “telemetry”, “haemoglobin”, “pregnancy”). Psychologists, instead, tended to use terms referring to a more general appraisal of patients' conditions as characterised by “deterioration”, “alcohol consumption” leading to a feeling of “concern”, but also by the appreciation for “progress” and “goals” that patients were pursuing in their journey. Further to this point, psychologists’ discharge letters tended to refer to “goals” and future directions when informing suggestions or more direct requests for patients to access additional support upon discharge.

### 3.2 Sentiment analysis

Based on the results from topic modelling, the next step was to use sentiment analysis to investigate how the two health professional groups' different choices of words and approaches signaled the “emotional tone” underpinning their interactions. The section below presents the tone variation between professional groups (psychologists and neurologists) and among document types. The previously mentioned pre-trained sentiment analysis models (VADER and Flair) were used to cross-validate the sentiment scores. A subset of patient records was analysed by domain experts to validate the tone of the patient records, and the results across letter types were implemented to further verify the model's sentiment output. It is important to note, in this regard, that the terms “positive”, “neutral” and “negative” emotional tone are used to refer to the overall emotional tone expressed within the text, essentially analysing whether the general sentiment conveyed by healthcare professionals in their letters is favourable, unfavourable, or neutral (87).

#### 3.2.1 VADER sentiment analysis

**Figure 4:**
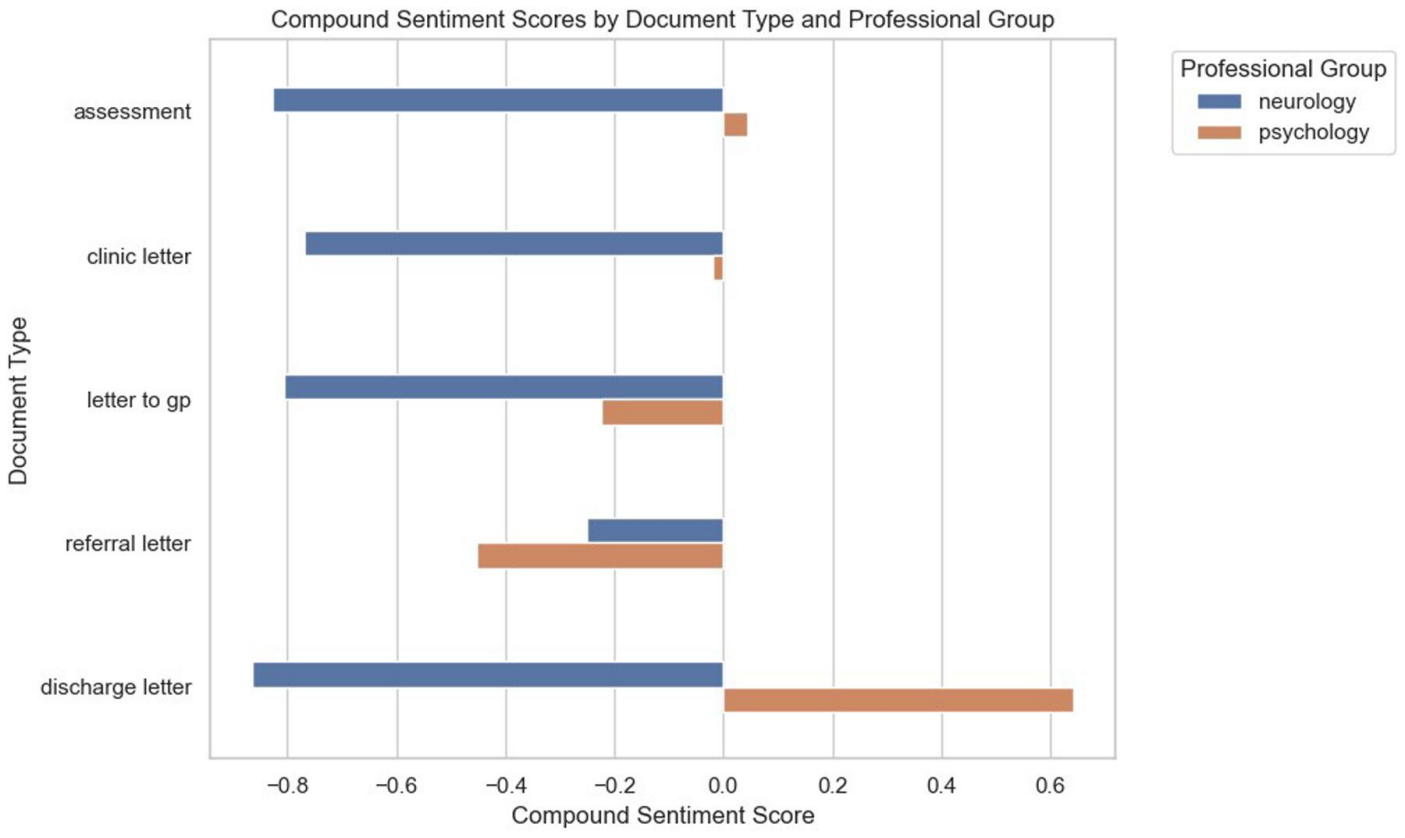
VADER-based compound sentiment scores between psychologists and neurologists across different letter types.

The compound sentiment scores across the two professional groups according to letter types are presented in Figure 4. Based on the plots, it can be inferred that neurologists' interactions with patients across all the stages of care tend to be quite cautious, which could explain the pronounced negative score. For psychologists, assessments and clinic letters tend to be more positive compared to GP letters and referral letters, with discharge letters showing a high level of positivity.

**Figure 5:**
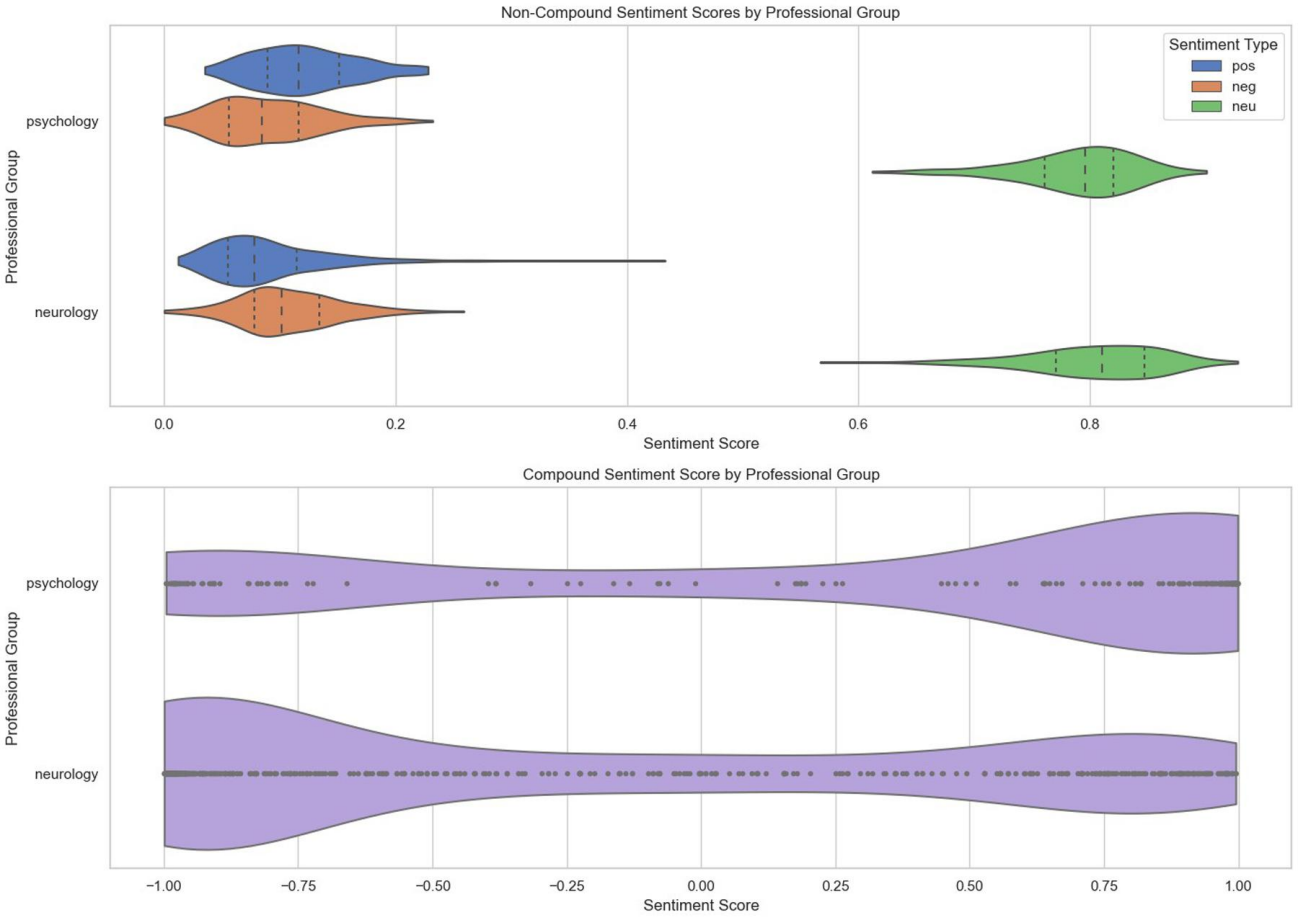
VADER-based compound and non-compound sentiment score distribution across professional groups only.

This was reflected when looking at the compound sentiment score distribution of the professional groups through the violin plots in Figure 5. Psychologists seemed to take a more positive, friendly, and informal approach than neurologists, with the bulk of their records leaning towards the positive side. Looking at the non-compound sentiment scores, it seemed that both psychologists and neurologists tended to use neutral sentiment keywords, with similar distributions of positive and negative sentiment keywords.

#### 3.2.2 Flair sentiment analysis

**Figure 6:**
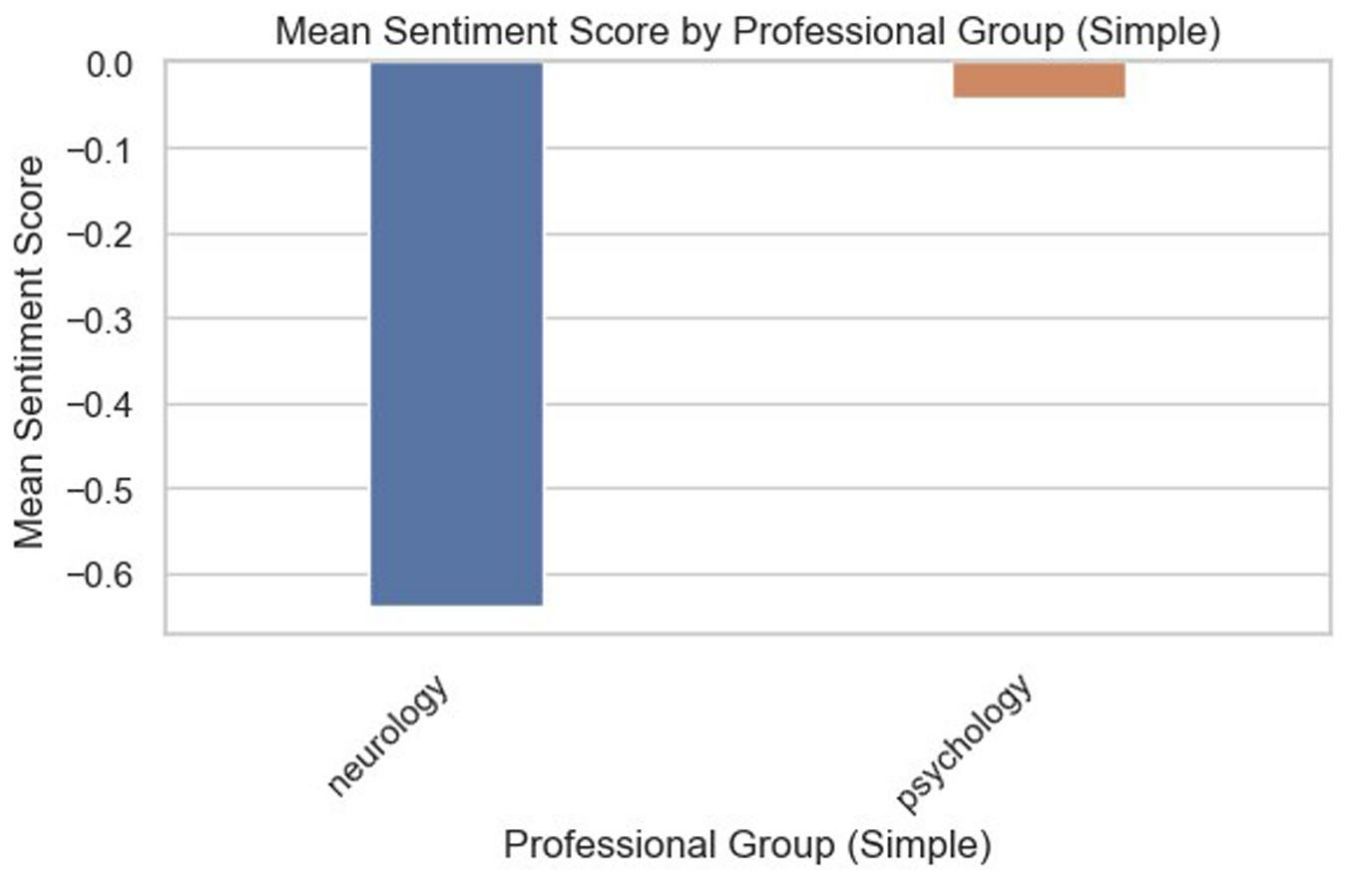
Flair-based sentiment scores across the two professional groups.

**Figure 7:**
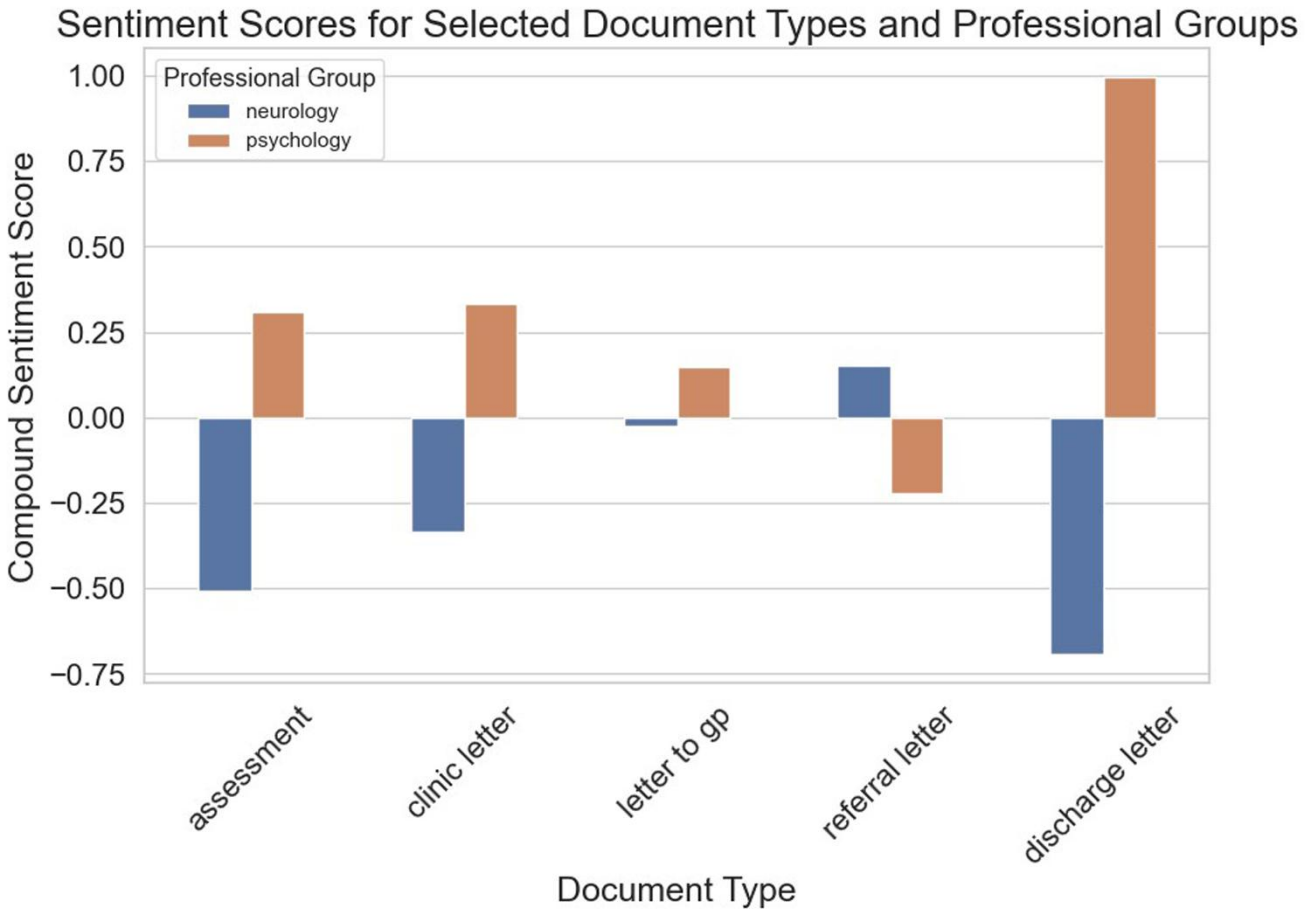
Flair-based sentiment scores across the two professional groups by letter types.

To cross-validate our sentiment results from VADER, the Flair model was also used to generate sentiment scores, as seen in Figures 6, 7. The results showed similar outcomes to our findings derived from the VADER model. As seen in Figure 7, psychology notes contained more positively valenced terms overall, while neurology notes showed a higher proportion of negatively valenced phrases. Inspection of exemplar sentences indicated that negative polarity often reflected clinical reasoning processes—such as documenting absent symptoms, reporting risk, or describing acute events—rather than interpersonal tone. Accordingly, these findings should be interpreted as differences in lexical valence rather than communicative style.

This is further reflected in Figure 6, referring to the analyses of patient records across the two professional groups. Overall, neurologists tend to be more critical and reserved in their approach when dealing with FND patients.

### 3.3 Expert review

To evaluate the performance of the sentiment model for medical note analysis, an expert validation was conducted to compare automated outputs with professional clinical judgement. Approximately 100 clinical documents were selected as a “ground truth” set, evenly drawn from texts the model had classified as neutral (*n* = 34), positive (*n* = 33), and negative (*n* = 33). A healthcare professional (DDB, expert clinical psychologist) who was blind to the model's results independently rated each document as positive, negative, or neutral based on tone and content. The expert's classifications were compared with the sentiment outputs of the VADER model using both the compound sentiment score and the non-compound percentage of positive, negative, and neutral words per document.

As shown in Table 3, there was a 61% agreement between model and expert ratings based on the compound sentiment score. Among the remaining 39%, most discrepancies involved one-degree differences—for example, model-rated negative vs. expert-rated neutral, or model-rated positive vs. expert-rated neutral. Seventeen of these mismatches fell within the “negative–neutral” range, the majority of which were neurologist documents (11 of 17). Similarly, of the 13 records in the “positive–neutral” range, most were also authored by neurologists (11 of 13). Only nine records exhibited two-degree differences (e.g., model rated positive, expert rated negative), eight of which involved expert-assigned negative ratings, again predominantly from neurology notes (8 of 9).

**Table 3:** Expert review results.

Metric	Observation
Compound sentiment score	61 matches
One-degree difference (neg to neutral or neutral to neg)	17
One-degree difference (pos to neutral or neutral to pos)	13
Two-degree difference (pos to neg or neg to pos)	9

Several factors may explain these divergences. Most notably, the VADER model was not originally trained on medical terminology or clinician–patient discourse and therefore does not account for contextual cues that influence how sentiment is expressed in clinical language. For instance, a document describing “some improvement” amid ongoing severe symptoms may contain positive lexical items but would likely be interpreted by a clinician as neutral or even pessimistic in overall tone. Clinicians are attuned to the broader narrative and clinical implications—such as disease severity, patient distress, or treatment uncertainty—that automated models cannot yet capture. Furthermore, the higher rate of mismatches in neurology documents suggests that more medicalised, problem-oriented language may be especially challenging for general sentiment algorithms to interpret accurately.

Interpreting sentiment within clinical documentation requires careful consideration of how linguistic polarity interacts with professional discourse and communicative intent. In contrast to everyday language, where negative sentiment typically implies disapproval or unfavourable evaluation, negative polarity in medical writing often reflects problem-focused or risk-oriented communication. Neurologists' documentation frequently included negatively weighted expressions describing clinical complexity, diagnostic uncertainty, or symptom deterioration—for example, “frequent seizures,” “poor response,” or “worsening episodes.” Such phrasing denotes the seriousness of the clinical presentation, rather than a negative attitude or bias. By contrast, psychologists' documentation more commonly contained positively valenced language emphasising progress, engagement, and adjustment, reflecting a therapeutic and recovery-oriented focus.

The clinician-guided review of sentiment contributors (Section 2.2.4) provided an additional interpretive layer, enabling evaluation of model reliability and contextual meaning. This review confirmed that most sentiment variation corresponded to disciplinary orientation and communicative purpose, not affective or moral judgement. Nonetheless, a small subset of terms carried evaluative or interpersonal connotations—for example, “noncompliant,” “attention-seeking,” or “difficult.” Although infrequent, such expressions are linguistically and ethically salient because they imply implicit assessments of patient character or behaviour. Prior research has shown that such phrasing can contribute to perceived disrespect, reduced empathy, and diagnostic overshadowing (88, 89, Fiske et al., 2019).

Accordingly, while sentiment polarity alone should not be interpreted as a direct indicator of stigma or clinician attitude, it provides a useful quantitative index of tone and communicative focus. When integrated with thematic and qualitative analysis, sentiment modelling offers insight into how professional discourse varies across disciplines and how these linguistic differences may shape the portrayal of patients within the health record. In this sense, sentiment analysis—when accompanied by expert clinical interpretation—can serve as a diagnostic and reflective tool for identifying patterns in documentation that merit further discussion in clinical communication training and practice (66).

## 4 Discussion

The results of this study provide exploratory insights into the linguistic and tonal differences between neurologists and psychologists when addressing FND patients in written clinical documents. By leveraging NLP techniques and sentiment analysis, the study highlighted how these professional groups differed in their clinical discourse, which may have direct implications for patient care, communication, and treatment approaches. This discussion will interpret these findings in light of their clinical relevance, theoretical implications, and potential for future research and practice.

### 4.1 Topic modeling: thematic differences between psychologists and neurologists

The topic modeling analysis revealed clear distinctions in the focus of communication between psychologists and neurologists, which underscores the different professional orientations of these two groups. Psychologists predominantly focused on themes related to emotional and subjective experiences, personal care, and the long-term management of symptoms. This is evident in the frequent use of words like “stress”, “awareness”, “feeling” and “support”, which reflect a holistic and patient-centred approach. This finding aligns with the role of psychology in FND, which is not only to diagnose and treat this condition, but also to address how patients manage their symptoms in their everyday life, help them to improve their overall mental health and quality of life, and decrease medical service utilisation (90–94). On the other hand, neurologists used more medical and technical language, with their communication centring on clinical and diagnostic terms such as “seizure”, “medication”, “assessment” and “history”. This is reflective of the medical model that neurologists employ in their treatment of FND, where the emphasis is on identifying physiological abnormalities and managing symptoms through clinical interventions (95). This also aligns with evidence on neurologists' pivotal role in diagnosing FND, explaining its mechanisms to patients and suggesting appropriate medical treatment to reduce the burden of FND symptoms (96, 97).

In other words, the findings of the present study seem to suggest that neurologists' communications are more likely to emphasise the patient's immediate clinical state and medical treatment, while psychologists take a broader view that encompasses emotional, psychological, and social factors. Importantly, this divergence in focus may reflect differences in training, professional responsibilities, and expectations placed upon these healthcare professionals. This finding has implications for interdisciplinary collaboration, as it suggests that optimal care for FND patients requires the integration of both perspectives: the neurological focus on symptom management and the psychological attention to emotional support and coping mechanisms.

### 4.2 Document types and professional communication styles

The analysis of specific document types (referral letters, clinic letters, GP letters, assessment letters, and discharge letters) shed further light on the granularity of communication differences between the two groups. Across all document types, psychologists tended to focus on a broad array of factors influencing FND patients, including psychosocial aspects such as childhood trauma, quality of life, and emotional coping strategies. Neurologists focused more narrowly on clinical symptoms, diagnosis, and medical treatment plans. For example, in referral letters, neurologists' communications were more clinical and diagnostic, with references to seizure types, medical history, and medications. Psychologists, by contrast, incorporated a broader understanding of the patient's life, discussing personal goals and emotional factors such as pain, stress, and sleep quality. This suggests that psychologists may be more likely to take a holistic view of the patient when communicating with other professionals, while neurologists may focus on ensuring that other clinicians are aware of the patient's medical condition and treatment needs.

More generally, the neurologists' tendency to use medical and diagnostic-centred communication and psychologists' proclivity to adopt a biopsychosocial perspective on assessment and care for FND was evident across all types of documents analysed. This calls for effective interdisciplinary collaboration to ensure that patients receive comprehensive care that addresses both their medical and psychological needs (98, 99). A failure to appreciate the complementary nature of these communication styles could lead to fragmented care, with one aspect of the patient's experience being overlooked (100). This integration in domain knowledge and communication styles appears to be even more relevant when considering that for the past half-century, the clinical management of FND has been subject to a great deal of fragmentation, with a paucity of care pathways mutually agreed upon between neurologists and psychiatrists (101). Besides being potentially detrimental to FND patients, this fragmentation often leads to poor outcomes and frustrations for both patients and professionals (102). In light of these considerations, studies such as the current one offer support for the need for a more substantial shift in these care models and the consequent recognition of the need for truly multidisciplinary and integrated care (103, 104).

### 4.3 Sentiment analysis: tone and emotional engagement

The sentiment analysis results provided another layer of insight into how psychologists and neurologists differ in their interactions with FND patients. Across all stages of care, neurologists tended to have a more cautious and often negative tone, while psychologists exhibited a more positive and emotionally supportive tone. This difference is particularly salient in the context of patient care, as the tone of communication can significantly impact a patient's experience, trust, and engagement with their treatment plan (105–107).

The positive language in psychological documents, such as “opportunity”, “progress” and “support”, may help to foster a more hopeful and proactive patient mindset, which evidence increasingly suggests can be particularly important in the management of chronic conditions like FND (108, 109). Conversely, the more negative tone found in neurologists’ documents may reflect the cautious and often uncertain nature of medical diagnosis and treatment in FND, a condition that is still not fully understood and can be challenging to treat (21, 110).

Neurologists may also focus more on risk factors, complications, and the need for medical interventions, which could explain the prevalence of negative sentiment. While this cautious tone is likely a reflection of the complexities and challenges of managing FND, it may also have unintended consequences for patient engagement and satisfaction. For instance, patients may feel discouraged or less hopeful if their interactions with neurologists are perceived as overly negative or detached. This datum appears to be even more important when applied to FND, as there is abundant evidence regarding the role that problematic conversations and stigmatising attitudes from clinicians can have on FND patients' disease management, access and experiences of care (111–113).

The differences in sentiment between the two professional groups also raise important questions about how tone influences patient outcomes. Research in healthcare communication has shown that positive, supportive language can improve patient satisfaction, adherence to treatment, and even health outcomes (114–116). In contrast, negative or overly cautious language may contribute to patient anxiety, dissatisfaction, and disengagement (113, 117, 118). Therefore, these findings suggest that neurologists may benefit from incorporating more positive and empathetic language into their communications, particularly when discussing treatment options and prognosis with patients.

Nonetheless, when interpreting differences in polarity and salient lexical items between neurology and psychology documents, it is important to recognise that sentiment analysis tools such as VADER and Flair capture lexical valence and intensity rather than interpersonal tone, stylistic nuance, or communicative stance. Negative sentiment values in clinical notes are frequently driven by documentation practices—such as describing absent symptoms, risk events, or acute presentations—rather than by affective tone or critique (50). Likewise, differences in wording between professional groups may reflect the distinct clinical purposes, encounter structures, and documentation norms of neurology and psychology encounters (119). For example, neurologists often document acute episodes, diagnostic uncertainty, and exclusionary reasoning, while psychologists typically describe ongoing therapeutic engagement, social support, and behavioural formulations. These contextual factors likely contribute to the observed polarity patterns and should caution against interpreting sentiment differences solely in terms of “communication style.” Accordingly, our interpretation of sentiment results is exploratory and should be understood within the constraints imposed by clinical workflows, note purpose, and the methodological limits of automated sentiment analysis.

In conclusion, while it is unsurprising that psychologists focused more on psychosocial themes and neurologists on diagnostic and medical detail, these distinctions offer important insights into how patients are represented and experienced in clinical documentation. The ways in which clinicians describe symptoms, progress, and interactions carry implications for both therapeutic framing and perceived patient legitimacy. Across both professional groups, we found that negative sentiment was usually associated with clinical challenges—for example, worsening symptoms, complex presentations, or limited response to treatment—rather than overtly pejorative tone. However, a small subset of phrases revealed more evaluative or interpersonal language, such as references to patients being “difficult,” “frustrating,” or “non-compliant.”

These terms, though rare, are meaningful because they can signal emotional distancing or frustration, which may inadvertently shape therapeutic dynamics and contribute to feelings of invalidation among patients. Similar findings have been reported in clinician documentation research: for example, in their qualitative study, Park et al. (88) found that physicians express negative attitudes via subtle language such as questioning patient credibility or labelling patients as “difficult.” In a separate study, Brooks et al. (89) documented that stigmatising language in hospital records was more common among Black patients and was associated with higher rates of diagnostic process errors. By contrast, positive sentiment in our corpus frequently co-occurred with language denoting collaboration, engagement, and improvement, such as “working well,” “engaged in therapy,” and “showing progress.” This suggests that tone can subtly mirror clinicians’ perceptions of therapeutic alliance and patient responsiveness.

Taken together, these observations indicate that linguistic tone and topic emphasis in FND documentation do more than reflect professional focus—they offer a window into clinicians' relational stance and the broader culture of care surrounding this condition. Identifying and reflecting on such patterns may support more balanced, patient-centred documentation practices and encourage awareness of how everyday language in clinical notes can influence patient experience and interdisciplinary understanding.

### 4.4 Expert review and model validation

The expert review of the sentiment analysis provides a critical reflection on the limitations of using NLP models like VADER and Flair in clinical contexts. The relatively low match (61%) between the model's sentiment classification and the expert's ratings highlights the challenges of applying general sentiment analysis tools to specialised medical documents. One key issue is that these models are not trained to understand medical terminology or the nuanced ways in which healthcare professionals discuss patients' conditions. For example, terms that the model may interpret as negative, such as “seizure” or “disorder”, may not necessarily carry a negative connotation in the context of a clinical document, where they are part of the routine language used to describe medical conditions. Likewise, positive terms like “progress” might not fully capture the severity or complexity of a patient's condition, leading to potential discrepancies between the model's output and the expert's interpretation.

There is increasing interest in using NLP to analyse textual information from clinical records. NLP has been successfully implemented in the FND domain, for example, to aid in accurately differentiating epileptic seizures from psychogenic seizures. Nonetheless, subsequent manual analyses are often required to supplement the NLP-generated outcomes with essential details and context, as NLP can fail to detect all nuances in text (120).

This suggests the need to develop specialised NLP models specifically trained on medical documents and to account for the need for expert input to provide details and interpretations that NLP analyses alone may not be able to capture, thereby improving the accuracy of NLP output and its effectiveness in aiding clinical practice and clinical decision-making processes. Lastly, future NLP applications in clinical practice (including, but not limited to, FND) would need to account for the specific context in which clinical language is used and understand the implicit meanings that healthcare professionals attribute to certain terms and phrases. Additionally, further research could explore how integrating domain-specific knowledge into NLP models enhances their ability to capture not only sentiment but also the intent and nuance of clinical communications.

## 5 Limitations

Several limitations of this study should be acknowledged. First, clinical documentation provides an indirect and partial representation of communication between clinicians and patients. The tone and phrasing in written records do not necessarily reflect how clinicians interact with patients during consultations. Clinical notes are shaped by institutional norms, time constraints, and medico-legal requirements, and may prioritise diagnostic clarity or brevity over interpersonal nuance. Consequently, while language in documentation can reveal aspects of professional framing and implicit attitudes, it cannot be assumed to mirror face-to-face communication style or empathy in practice.

Second, the NLP methods used (LDA) for topic modelling and lexicon-based sentiment analysis carry well-recognised methodological constraints. LDA assumes a “bag-of-words” representation and cannot capture word order or deeper syntactic and pragmatic relations, potentially oversimplifying the structure of clinical discourse. Similarly, sentiment models such as VADER were not trained on medical or psychotherapeutic language and may misclassify terms with clinical rather than emotional polarity (e.g., “poor control,” “severe episode,” “failure to respond”). Although these limitations were mitigated through domain-informed pre-processing and expert validation, the analyses nonetheless reflect approximate, rather than exhaustive, representations of meaning. Third, due to the need to protect patient confidentiality and anonymity, and in line with the ethical approval obtained for this study, no patient-level demographic or outcome data were collected or reported. As the aims of the present study were to analyse the content and emotional tone of clinician–patient communication, such demographic information was not required to address the research questions. Nevertheless, future studies employing NLP and sentiment analysis may wish to incorporate patient demographics, where ethically permissible, to examine whether communication patterns vary according to characteristics such as gender or age among individuals with FND.

An additional limitation concerns the disciplinary backgrounds of the research team involved in interpreting the NLP outputs. The clinical psychologist (DDB) contributing to topic and sentiment interpretation is most familiar with psychological documentation norms and less familiar with the specific linguistic, structural, and workflow conventions typical of neurology notes. Although interpretive judgements were reviewed collaboratively with a neurologist (RM) and a data scientist (MSM) experienced in analysing neurology documentation, cross-specialty comparisons may still be influenced by domain-specific expectations about how information is typically recorded. Prior work has shown that documentation practices vary substantially across clinical specialties due to differences in encounter purpose, diagnostic reasoning patterns, and narrative traditions As such, observed differences in sentiment or lexical patterns between neurology and psychology should be interpreted cautiously, and understood as potentially reflecting not only communicative tendencies but also specialty-specific documentation norms.

Finally, the data were drawn from a single UK hospital, and the findings may not be fully generalisable to other healthcare systems or documentation cultures. Language use is influenced by institutional style, professional training, and national healthcare context. Replication across other sites, including community and international settings, will be required to test the robustness and transferability of these findings.

Despite these limitations, the study provides a novel empirical foundation for examining professional discourse surrounding FND through the lens of natural language processing. Future work could integrate patient-reported experience measures, apply context-aware deep learning models capable of capturing semantic nuance, and explore cross-institutional comparisons to extend both the interpretive depth and generalisability of the present findings.

## 6 Implications for practice and future research

The findings of this study have several important implications for clinical practice and future research. First, the clear differences in communication styles between psychologists and neurologists suggest that interdisciplinary teams must be mindful of these differences when coordinating patient care. Neurologists and psychologists should aim to communicate more effectively, ensuring that both the medical and psychosocial aspects of a patient's care are given equal attention. Regular interdisciplinary meetings and joint case discussions could help bridge this gap and ensure that patients receive holistic, integrated care (41, 121, 122).

Second, the sentiment analysis results suggest that neurologists could benefit from adopting more positive and supportive language when interacting with patients. This could be particularly important in cases where patients are struggling with the emotional and psychological burden of FND, and where a more empathetic and hopeful approach may improve patient engagement and outcomes.

Future research should explore how these communication differences and sentiment patterns may influence patient outcomes. For example, it would be useful to determine whether patients who receive more positive and supportive communications from their healthcare providers report higher levels of satisfaction or adherence to treatment or if different communication styles may lead to differences in clinical outcomes. More generally, understanding the impact of communication on patient care in more detail could provide valuable insights for improving interdisciplinary collaboration and enhancing patient outcomes in FND management.

## 7 Conclusion

While exploratory in scope, this study contributes to an underdeveloped evidence base on the use of AI-driven text analysis to examine clinician–patient communication in FND. By integrating computational techniques with clinician-led interpretation, it demonstrates the potential of NLP methods to enrich future interdisciplinary inquiry into healthcare language.

## Data Availability

The data analyzed in this study is subject to the following licenses/restrictions: the data that support the findings of this study are available from Northern Care Alliance NHS Group Trust (approval number 22HIP47) but restrictions apply to the availability of these data due to the nature and sensitivity related to patient records, which were obtained and used with relevant guidelines from the Research & Innovation (R&I) department of the Northern Care Alliance NHS Group Trust for the current study, and so are not publicly available. Requests to access these datasets should be directed to d.dibasilio@lancaster.ac.uk.
