# Supplementary material for: Using natural language processing to explore differences in healthcare professionals’ language on Functional Neurological Disorder: a comparative topic and sentiment analysis study
Source: Front Digit Health. 2026 Jan 16;7:1691724. doi: 10.3389/fdgth.2025.1691724 (PMC12855439; doi:10.3389/fdgth.2025.1691724)
Supplement: Supplementary file 1 [file Supplementaryfile1.docx]

**Appendices**

**Appendix 1 - Types of clinical documents collected and analysed**

| **Document type** | **Rationale and purpose** |
| --- | --- |
| **Referral letter** | Letter from neurologists to neuropsychologist referring for therapy input following confirmation of diagnosis of FND |
| **Clinic letter** | Letter summarising consultation in neurology and neuropsychology clinics sent to referring clinicians (e.g., primary care), usually copied to other clinicians involved. |
| **GP letter** | Letter sent to primary care physicians from neurology and neuropsychology departments. Usually relates to results of any investigations arranged from the clinic (e.g., EEG, MRI). |
| **Assessment letter** | Letter sent from the neuropsychology department following initial attendance at the clinic, addressed to the referring neurologist and primary care. |
| **Discharge letter** | Letter summarising a period of inpatient assessment and treatment, usually relates to inpatient video telemetry for diagnosis of FND. |

**Appendices 2 to 6 – Most salient topics and related keywords identified across document types in the two professional groups (psychologists and neurologists) considered**


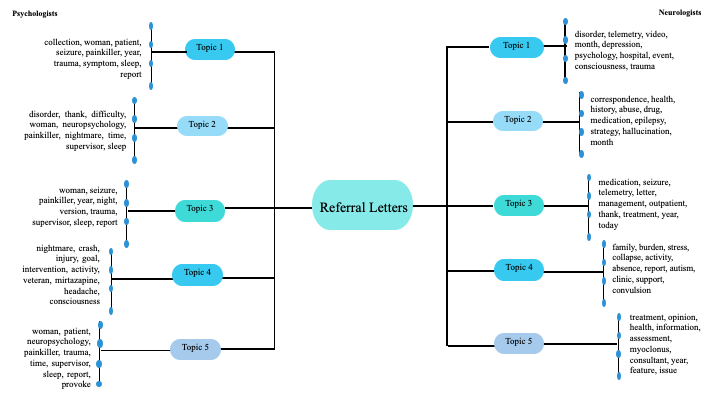
**Appendix 2** – Referral letters


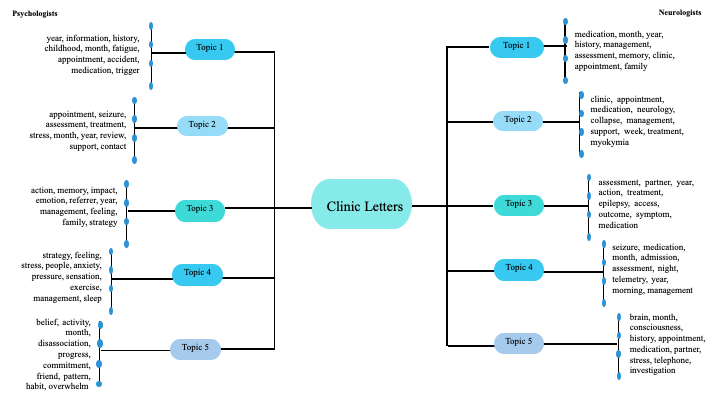


**Appendix 3** - Clinic letters


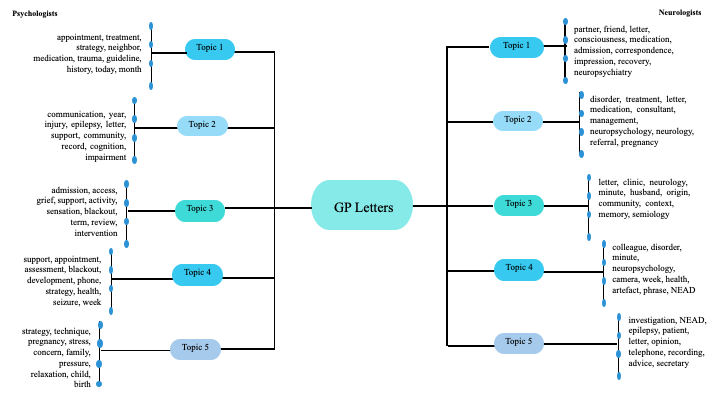


**Appendix 4** – GP letters


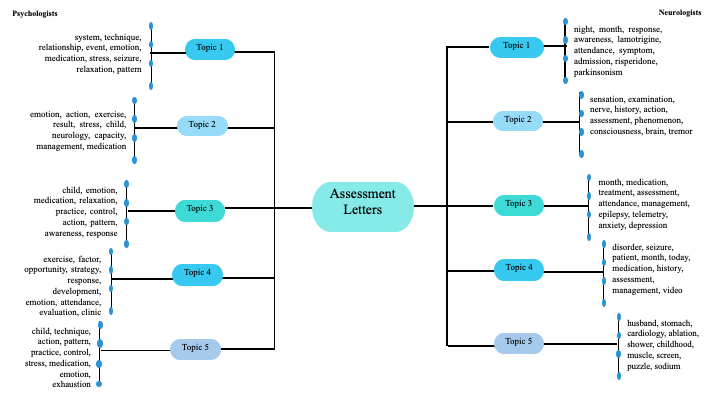


**Appendix 5** – Assessment letters


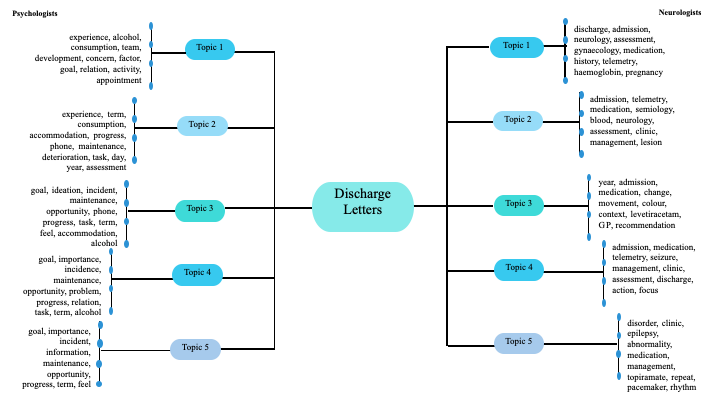


**Appendix 6** – Discharge letters

**Appendix 7**

Final chosen model parameters for choice of 5 topics for both psychology and neurology dataset

- num topics = 5
- *α* = 0*.*01
- update every = 1
- chunksize = 100
- passes = 15
- per word topics = True
